# Diseases of the Colon

**Published:** 1910-09

**Authors:** 


**Diseases of the Colon.** By P. Lockhart Mummery, M.B., B.C., F.R.C.S. Pp. vi, 322. Bristol: John Wright & Sons Ltd. 1910.

This very excellent little book is written by one who for many years past has devoted special attention to that branch of surgery which deals with diseases of the colon and rectum. It presents the subject in a very lucid fashion, and there are enough clear case reports to make the descriptive matter vivid without over-burdening it with detail. It will serve rather as a source of reference for general practitioners than as an exhaustive monograph. The chapters on colitis, and the description of the use of the sigmoidoscope are the most valuable in the book, and it is noteworthy that the author attaches a very high importance to the operation of appendicostomy, whereas that of ileo-sigmoidostomy is kept much in the background. In the description and treatment of chronic constipation Mr. Mummery adheres to safe conservative principles, and says but little in favour of heroic measures.

The chapters on anatomy, physiology and malignant disease do not seem to be as complete as the importance of these subjects demands. When it is stated that the average length of the colon is twenty-two inches (page 2) we presume that the transverse portion only is referred to. The description of certain methods of treatment is not sufficiently detailed to be of any use (*e.g.* electrical treatment on page 103).

We think that the value of the book would be greatly increased if a larger reference were made to the very considerable continental literature on the subject, and also if a more complete list of authorities and papers was added to each chapter.

